# Assessment of users' adoption behaviour for stock market investment through online applications

**DOI:** 10.1016/j.heliyon.2023.e19524

**Published:** 2023-08-29

**Authors:** Amar Johri, Mohammad Wasiq, Harpreet Kaur, Mohammad Asif

**Affiliations:** a College of Administrative and Financial Sciences, Saudi Electronic University, Riyadh, 11673, Saudi Arabia; b Minerva Institute of Management and Technology, Dehradun, India

**Keywords:** Stock market, Online applications, User behaviour, Investment: stock trading

## Abstract

Investing in stocks has become increasingly accessible, with beginners able to start an account via a brokerage's website or mobile app with very little money. Online trading through applications allows you to trade independently without a broker's interference. This not only lowers the overall cost of trading but also makes it hassle-free, increasing the value of the business significantly. Using an online trading account gives an investor more control. This paper examined the user's perspective on stock market investment through online applications. The impact of users' awareness, benefits & choice of investment, reliability, safety, risk-related factors, financial literacy, technical aspect, and dependency was analyzed on users' adoption behaviour. An empirical survey with a valid sample of 424 respondents from India was collected from the respondent and analyzed using descriptive analysis, correlation analysis, reliability analysis, analysis of variance, and regression analysis. Results indicated that users' awareness, benefits & choice of investment, reliability, safety, risk-related factors, financial literacy, technical aspect, and dependency significantly influence the users' adoption behaviour in using stock trading applications. The findings will assist potential investors in comprehending the factors influencing the adoption of such apps and aiding the organizations engaged in operating or creating such applications in understanding user behaviour, which will significantly add value to the society at large.

## 1 Introduction

The stock market has developed a lucrative investment platform for investors. The stock market has grown progressively, grabbing investors' attention. The stock market continues to be an efficient and attractive platform for individuals looking to increase their wealth in the ever-changing financial landscape. In the past, buying and selling stocks needed extensive knowledge, skill, and communication with stockbrokers. However, the rise of online applications brought about by the introduction of technology has revolutionized the investment scene and made it more approachable for the common population. The stock market is where buyers and sellers meet to exchange equity shares of the public corporation. There are various stock market investment methods, such as offline and online investments. The web and the internet together create a platform for e-commerce transactions. E-commerce provides a platform for computer users to find product and service information and complete online buying and selling. In addition, M-commerce is the subset of e-commerce that allows mobile users to access product and service information and complete online buying and selling [1]. Online trading is an internet environment infrastructure to organize stock exchange on the website using a few stock exchange websites [2].

The mobile platform allows running stock trading applications by investors to search for and trading of stocks quickly. The mobile platform can access the internet from various mobile devices such as tablets and smartphones [1]. The primary means of reaching the internet worldwide is through highly portable devices such as smartphones and tablets, not traditional desktop or laptop PCs. The mobile platform provides various numbers of mobile applications for the stock market. Leading companies such as Upstox, ICICIdirect Market, Angle Broker, Zerodha Kite, Motilal Oswal and 5paisa online trading applications provide mobile applications for customers to invest in the stock market. A stock market application is an online platform that makes it easier to buy and sell on the trading platform and update and manage investments. Online stock exchange conducting stock exchange through various mobile applications. An online exchange application is a mobile application through which investors can see the commercial transaction of stocks and other assets online.

The mobile technology platform is the latest and most advanced development in internet infrastructure that permits on-demand access to information on the mobile web browser and social media from wireless devices such as mobile phones, personal digital assistants and smartphones, and tablets. The on-demand access has penetrated the capital market; it is found in the latest report that buyers and sellers depend more on mobile internet technology for communication, trading and analysis. For instance, one-third of Vanguard customers often access their trading accounts through mobile devices [3]. Mobile online technology permitted stock market players to access wirelessly and analyze and trade financial product information in real-time. This productive approach to accessing the stock market information via mobile internet may lead to lower search costs, trading costs, and information dissemination costs [4,5]. The Indian share market, in 2021, touched the $ three Trillion milestone. According to the financial market, experts' reasons for this progress in the member of investors is the emergence of new technology. Online mobile application trading and investment have considerably transformed the Indian stock market. This study aims to examine stock market investment through the online trading application.

### 1.1 Problem statement

The rapid growth of technology has revolutionized the financial landscape, which has made stock market investment more accessible and convenient through online applications. Nonetheless, despite the growing popularity of these platforms, understanding user adoption behaviour remains a crucial challenge for financial sector players. The issue is understanding the elements impacting consumers' decision-making processes and the barriers they confront when investing in the stock market through online applications. The motivation behind the present study was to understand the main factors influencing users' adoption of online applications for stock market investment.

In conclusion, the purpose of this study article is to provide a complete assessment of consumers' adoption behaviour for stock market investment via online applications. We hope to pave the path for a more educated and inclusive investment landscape, increasing the potential for financial growth and empowerment in the digital era, by investigating the drivers and barriers impacting users' decisions.

## 2 Literature review

### 2.1 Awareness

The primary responsibility of stock market players is to create awareness among stock market investors. Awareness of financial markets leads investors to select the desired investment decision and to avoid risk. The authors pointed out a need for buyer awareness due to ideas of funding patterns. Authors have also revealed that certain factors are essential in investors' thinking about their investment, such as age, gender, income, education, and socio-financial variables [6]. The authors analyzed and concluded that three constraints are significant. These three factors cannot be avoided, such as stock market investment, stock market awareness, and stock market information are essential for investors. If the investors do not have adequate information on stock market variables in a specific field will direct the massive loss. Investors should have adequate knowledge of identified factors that will guide them to make the right decision to flourish in the stock market [7]. Based on the previous discussions, we can extract the following hypothesis.

H1 The adoption behaviour is influenced by users' awareness of such applications.

### 2.2 Benefits and choice of investment

According to the author, investment in the stock market is risky and doubtful. Individuals capitalize their income into available alternatives, which has numerous benefits and leads to reducing the risk. For that cause, every individual has considered three fundamental aspirations when investing in the stock market: to safeguard the risk, boost the wealth and maintain the liquidity [8]. The author found that discovering the right stock is the most significant to any investment strategy and commercial transaction. The choice of the right stock depends on the individual interested in the stock market's transactions. The investment choice is also related to the time factor, i.e., the investor is interested in long-term or short-term investment [9]. The authors identified foreign portfolio investors as preferably holding large stocks in large-cap firms and the service industry, domestic institutional investors as preferable to holding vast numbers of shares in the paper industry, and retail investors who preferably hold considerable shares in the textile and chemical industry. Foreign portfolio investors choose those firms which are registered on a foreign market. Domestic investors, particularly retail investors, favor small-cap stocks, and those firms' procedures need local knowledge in addition to that industry diversification direct investment decision. Government guidelines and modifications are essential in attracting investors [10]. Along with all other factors, demographic characteristics also significantly influence investors' behaviour in adopting online trading. The adoption of Internet trading is influenced by factors like home ownership, income, trading expertise, and occupation. However, this decision is not significantly affected by factors like age, gender, educational attainment, the nature of the trade, and trading frequency. The adoption of Internet trading is not heavily influenced by perceived advantages or risks [11].

The advantages of online trading programs may significantly influence whether or not people choose to engage in online trading. Accessibility, ease, cost-effectiveness, security, dependability, mobile adaptability, and compatibility are ways the different benefits might affect the adoption of online trading. Overall, the variety of advantages provided by various online trading programs is vital in influencing consumers' opinions and choices. Higher adoption rates can be achieved by a comprehensive and alluring set of benefits; however, more features or security issues may be needed to improve the development of a specific platform.

According to researcher, social influence, perceived financial cost, expected performance, and perceived legitimacy all had major roles in determining individuals' intentions to adopt mobile banking [12]. The simplicity of use and quick access to financial markets via online platforms influence user intentions to engage in online trading. However, important considerations in Internet trading systems include perceived trust, usefulness and ease of use [13]. There is a substantial positive relationship between attitude, perceived behavioral control, reported benefits, and intention to engage in mobile stock trading[14]. Cost-effectiveness is particularly tempting to retail investors and might influence their decision to transfer from traditional brokerage services to online platforms. Based on the previous discussions, we can extract the following hypothesis.

H2 The users' adoption behaviour is influenced by the benefits and choice of investment of such applications.

### 2.3 Reliability, safety, and risk-related factors

The authors explained that any information stimulates stock prices when investors find the information is reliable and relevant [15]. The author examined that sustainability information required a certain level of reliability to guide the investors in identifying the company’s market value [16]. The authors found that perceived risk indirectly and directly impacted investment intention. It is also found that investors with a high-risk perception in stock investment, higher investments and perceived risk in investing enhance their higher investment decision [17]. Investors' investment decision has a strong association with risk, and Investors consider risk a central concept to analyze the investment decision [18]. Privacy risk and security can be defined as the threat and harm that reduce the service safety and individuals' investor concern about personal information [19]. Based on the previous discussions, we can extract the following hypothesis.

H3 Reliability, Safety, and risk-related factors significantly impact the users' adoption behaviour.

### 2.4 Financial literacy

A product is anything that could satisfy the needs and wants of human beings, and product knowledge is measured as a prominent thing in decision-making and information-processing research [20,21]. Financial literacy is a basic knowledge of financial investment concepts such as calculating interest rates, inflation, and risk diversification [22]. Customer investment behaviour is influenced by their financial knowledge [23]. It has been identified that there is a prerequisite requirement for customers to gain financial knowledge and awareness through attending seminars or financial education programs organized by several organizations. It is also found that objective financial literacy positively correlates with intention and knowledge [24]. Product knowledge enhances an individual’s decision-making ability [25] and reduces the dependency on information [26]. Self-assessed financial literacy was found to improve the likelihood of deciding on a stock market investment [27].

In the context of contemporary finance, the link between financial literacy and the acceptance of Internet trading is a crucial one. It is vital to remember that while financial literacy favorably promotes the adoption of online trading, it does not ensure trading success. Even those who are financially savvy should approach online trading cautiously and with an understanding of their risk tolerance and investment objectives because it still carries inherent dangers. Governments, academic institutions, and financial service providers frequently attempt to increase financial literacy through various initiatives and tools to enable people to make better financial decisions, particularly those about online trading.

The financially literate persons are more likely to use online trading platforms because they can better weigh their investing decisions' potential risks and rewards. Additionally, these investors are more likely to diversify their portfolios and choose well-informed investments, which improves trading results. Financial literacy plays a crucial role in facilitating educated decision-making processes pertaining to investments. [28]. People are deterred from online trading due to a lack of comprehension of financial words, market principles, and investing techniques. Additionally, a person with financial literacy has the knowledge and skills to manage their finances well, including budgeting, saving, and paying off debt [29]. Based on the previous discussions, we can extract the following hypothesis.

H4 Financial literacy impacts the users' adoption behaviour.

### 2.5 Knowledge of technical aspects

Mobile technology platforms allowed investors to perform investment activity to become more flexible, transparent, and faster. The technological invention has modified the financial market. The technological invention in the investment industry permits traders and investors to complete commercial transactions immediately while actively handling their financial portfolios from anywhere around the globe [14]. Online trading applications allow investors to make investment decisions about the financial market from anywhere, anytime, anywhere. Financial market investors have various technological fears in online investment, such as perceived risk, trust, and security in the system [3]. The financial market had transformed with the invention and use of internet facilities in stock trading investment [30]. There is various activity performed by online investors, such as the price of the stock and analyzing company information and stock performance by using their handheld device [31]. This study revealed that in line with a behavioral financial aspect, the amount of information is essential for investors [32]. Public information makes them more familiar with financial services and mobile application technology [33]. Authors found that public information such as credit scores, interest rates, and other investment details are essential in financial planning [34]. The financial service providers delivering the correct information will increase initial trust [35]. A new method of offering financial services globally has emerged as a result of technological progress. On the other hand, implementing electronic money comes with risks and challenges. Security, revenue and cost dimensions, and technological architecture all impact e-finance due to the advancement of global technology [36]. Based on the previous discussions, we can extract the following hypothesis.

H5 The knowledge of technical aspects influences the users' adoption behaviour.

### 2.6 Dependency

Customers' stock investment decisions depend on experience, age, and risk perception [37,38]. It is revealed in previous studies that important information plays a critical role in building the relationship between retail investors and human financial advisors [39]. Stock market investors must rely on public information to determine trust in financial technology solutions [40]. These days, social media are considered a reliable source of information for making financial decisions [41]. Social influences removed that investors' performance depends on others' feedback [42] and also found that customers tend to believe private information rather than public information or leaked information [43,44]. Based on the previous discussions, we can extract the following hypothesis.

H6 The users' adoption behaviour is influenced by the user’s investment dependency.

### 2.7 Adoption behaviour

The investors' behaviour in adopting online applications for e-trading and other investment decisions is affected by various factors. Some main factors influencing investors' behaviour are effort expectations, performance expectations, and perceived returns [45]. The perceived return and perceived risk were measured as significant forecasts of investors' adoption behaviour in the financial market [46,47]. Facilitating conditions positively influence the adoption intention of investors irrespective of the kind of online activity. Prior research revealed that facilitating conditions and technological innovation lead to the adoption behaviour of investors [48,49]. More significant revenue influences the stock market investors to invest in the financial market, and investors elect online platforms to generate and receive more revenue frequently [50]. Behaviour intention and facilitating conditions influence the adoption behaviour of investors through a mobile application for the online stock transaction and also found that future investors should consider these factors during mobile stock trading [45].

Based on the above literature review, the study identified the six variables to examine the impact on users' adoption behaviour which is presented in Table 1.

**Table 1:** Constructs of the study and its sources.

S.No.	Construct	Sources
1	Awareness	Umamaheswari and Kumar [6]; Prabhu and Gajendran [7].
2	Benefits and choice of investment	Ashraf and Baig [9]; Chhimwal et al.[10]);
3	Reliability, Safety, and risk-related factors	Fama et al. [15]; Ashraf and Baig [9]; Lackmann et al. [16]; Trang and Tho [17]; Pinasti et al. [18]; Al-Khalaf and Choe [19].
4	Financial literacy	Lusardi, and Mitchell [11]; Caroline et al. [23]; Mishra [24];
5	Technical aspect	Ancuta [30]; Bapat [34]; Chong et al. [14]; Nourallah [31];
6	Dependency	Koestner et al. [37]; Stålnacke [32], Florendo and Estelami [41]; Bommer et al. [43]; Chan et al. [44]
7	Adoption behaviour	Nouri et al. [46]; Chao [48]; Palau-Saumell et al. [49]; Rahman et al. [50]; Nair et al. (2022);

## 3 Objectives of the study

1. To find out the awareness level of people on online share trading applications.
2. To examine people's benefits and choice of investment towards investing in the stock market through online share trading applications.
3. To analyze the reliability, safety, and risk-related factors of investing in the stock market through online share trading applications.
4. To examine financial literacy and technical knowledge in the context of using online share trading applications.
5. To examine the investment dependency and adoption behaviour towards investing in the stock market through online share trading applications.

## 4 The research model

**Fig. 1:**
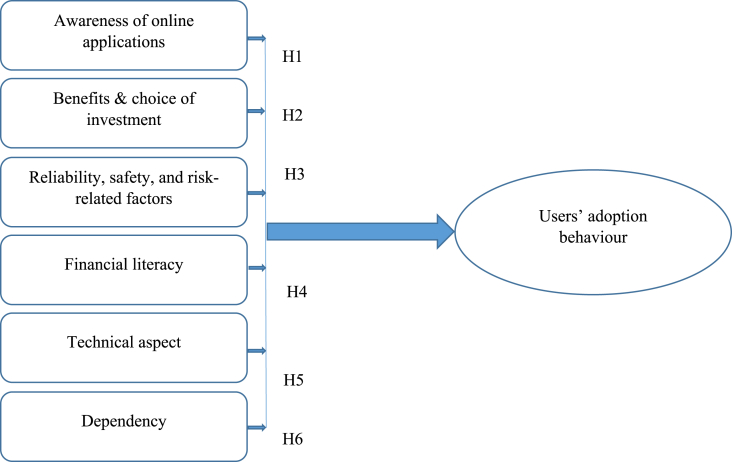
Proposed conceptual framework.

The study identified six independent variables: awareness, benefits & choice of investment, reliability, safety, and risk-related factors, financial literacy, technical aspect, and dependency. To explore the impact of these variables, the study defined one dependent variable, users' adoption behaviour. These factors formed the hypothesis and research model to measure the study's aims. Fig. 1 presents the proposed conceptual model of the study:

## 5 Research method

### 5.1 Data collection and sampling

This study employs a quantitative research methodology and is empirical. A standardized questionnaire with a 5-point Likert scale was employed to collect data from the respondents. An online survey was used to obtain the data. A total of 29 statement-based questions, categorized under seven constructs, about knowledge of online trading apps, rewards and investment choice, dependability, safety & risk-related variables, financial literacy, technical elements, dependence, and adoption behaviour were used in the questionnaire. The data was collected by convenient sampling method, a type of non-probability sampling. A pilot test of the questionnaire was conducted to assess the respondent's comprehension of the questions. In the pre-test, the responses of 50 individuals were observed. The pre-testing of the questionnaire contributed to the creation of the final questionnaire, and all items were retained in the online survey. A total of 424 individuals participated in the survey and offered important feedback. Informed consent was obtained from all participants in the study. The respondents were informed in the questionnaire about the confidentiality of the data and ensured the use of data for analysis purposes. However, ethics approval was not required for this study as the study was designed to explore the user's behaviour toward the stock market application through a quantitative approach.

### 5.2 Data analysis

To evaluate the data gathered for this investigation, SPSS was employed. Descriptive analysis, reliability tests, correlations, and regression analysis were used to examine the acquired data.

#### 5.2.1 Demographic profile

Table 2 outlines the demographic features of the respondents. 258 (60.85%) of the 424 respondents were male, compared to 166 (39.15%) female. Seventy-four respondents (17.45%) were under the age of 20 years, 166 respondents (39.15%) were between the ages of 21–30 years, 75 respondents (17.69%) were between the ages of 31–40 years, 45 respondents (10.61%) were between the ages of 41–50 years, 44 respondents (10.38%) were between the ages of 51–60, and 20 respondents (4.72%) were beyond the age of 60 years. Fifty-eight respondents (13.68%) were government employees, 185 (43.63%) were privately employed, 89 were entrepreneurs, and 92 (21.70%) were students. Two hundred fifty-five respondents had a monthly salary between Rs. 20,000 to Rs. 60,000. 278 respondents invested in the stock market, while 146 did not. The majority of respondents in both categories, those who trade and those who do not, would prefer to utilize an online trading application to invest in the stock market, which is an excellent indicator for analysis.

**Table 2:** Demographic profile of the respondents.

Variable	Category	Frequency	Percentage
Age	Less than 20	74	17.45
	21–30	166	39.15
	31–40	75	17.69
	41–50	45	10.61
	50–60	44	10.38
	Above 60	20	4.72
Gender	Male	258	60.85
	Female	166	39.15
Profession	Government Employee	58	13.68
	Private Employee	185	43.63
	Business	89	20.99
	Student	92	21.70
Monthly Income			
	Rs. 20,000–40,000	190	44.81
	Rs. 40,000–60,000	65	15.33
	Rs. 60,000–80,000	46	10.85
	Rs. 80,000–100,000	53	12.50
	Above Rs. 100,000	70	16.51
Do you invest in the stock market?	Yes	278	65.57
	No	146	34.43
If yes then, which one of the following modes do you prefer to invest in the stock market?	Financial Advisor	94	22.17
	Online Applications (Invest on your own)	330	77.83
If no then, which one of the following modes will you prefer to invest in the stock market?	Financial Advisor	84	19.81
	Online Applications (Invest on your own)	340	80.19

The mean and standard deviation for each variable was calculated. The mean value was obtained using the following formula.

μ = (ΣX)/n,where,

ΣX = Sum of all values in the dataset X,

“n” = Total number of observations.

Subsequently, the values of standard deviation for each of the six variables were obtained using the formula

σ = √[(Σ(X − μ) ^2^)/n],where

Σ(X − μ)^2^ = Sum of the squared differences between each value in the dataset (X) and the mean value (μ).

“n” = Total number of observations.

From the mean, standard deviation, minimum, and maximum values listed in Table 3, it is concluded that all six variables are essential to the users' adoption behaviour. On the other hand, the influence of awareness is the most substantial, followed by dependency and all other variables.

**Table 3:** Descriptive statistics of the variables.

	N	Minimum	Maximum	Mean	Std. Deviation
Awareness	424	2.5	5	3.774	0.620
Benefits and choice of investment	424	2.33	5	3.613	0.630
Reliability, Safety, and risk-related factors	424	2.6	5	3.615	0.529
Financial literacy	424	1.75	5	3.489	0.753
Technical aspect	424	2	5	3.268	0.799
Dependency	424	3	5	3.764	0.493

#### 5.2.2 Reliability test

Reliability analysis (Cronbach's alpha) was performed to examine the constructs' internal consistency. SPSS was used to assess each construct's reliability. The dependability of each concept and its interpretations are summarized in Table 4. Cronbach's Alpha value of each variable is more than 0.70. Cronbach's alpha was in the range of 0.732–0.874, which indicates that the data was highly reliable and internally consistent. This indicates that the scale used to gather the data was accurate and sufficient for the investigation.

**Table 4:** Reliability analysis of the variables.

Constructs	N	Number Of Items	Cronbach’s Alpha	Internal Consistency
Awareness	424	4	0.874	Excellent
Benefits and choice of investment	424	3	0.814	Excellent
Reliability, Safety, and risk-related factors	424	5	0.732	Excellent
Financial literacy	424	4	0.819	Excellent
Technical aspect	424	3	0.829	Excellent
Dependency	424	4	0.864	Excellent
Users' adoption behaviour	424	6	0.834	Excellent

#### 5.2.3 Correlation analysis

In order to investigate the degree and direction of association between two variables, correlation analysis is frequently performed. The correlation was calculated using the formula

r = (Σ [(Xi − $\bar{X}$)*(Yi − Ȳ)])/[√(Σ (Xi − $\bar{X}$) ^2)*√(Σ (Yi − Ȳ)^2)]where:

Xi and Yi = Individual data points for variables X and Y, respectively.
$\bar{X}$ and Ȳ = The means of variables X and Y, respectively.

Table 5 displays the association between the dependent and independent variables. According to the results of the correlation analysis, awareness of online applications r (424) = 0.29, p < .05), benefits & choice of investment r (424) = 0.52, p < .05), reliability, safety, and risk-related factors r (424) = 0.58, p < .05), financial literacy r (424) = 0.14, p < .05), technical aspect r (424) = 0.28, p < .05), dependency r (424) = 0.80, p < .05), and users' adoption behaviour were found to be significantly correlated.

**Table 5:** Correlation analysis of the variables.

	AW	BCI	RSRF	FL	TA	DEP	UAB	p value
**AW**	1							0.000
**BCI**	0.542170517	1						0.000
**RSRF**	0.458668476	0.529758510	1					0.000
**FL**	0.733348261	0.301510856	0.419126426	1				0.000
**TA**	0.728775145	0.383700153	0.483303337	0.785980502	1			0.000
**DEP**	0.399245069	0.320274811	0.489512473	0.353392160	0.342116339	1		0.000
**UAB**	0.292900242	0.523514319	0.587446730	0.149901954	0.284093503	0.801638813	1	0.000

**Correlation is significant at the 0.01 level (2-tailed).

#### 5.2.4 Bivariate regression analysis

This study includes a dependent variable (Users' adoption behaviour) and six independent variables (awareness of online applications, benefits & choice of investment, reliability, safety, risk-related factors, financial literacy, technical aspect, and dependency). Because of the likelihood of multicollinearity and the need for individual regression coefficient analysis, bivariate regression analysis was performed using the following formula:

Regression Model Specification:

UAB = β0 + β1 AW + β2 BCI + β3 RSRF + β4 FL + β5 TA + β6 DEP + ε

where:

UAB = Users' adoption behaviour
β1 AW = Awareness of Online Applications
β2 BCI = Benefits & Choice of Investment
β3 RSRF = Reliability, safety, and risk-related factors
β4 FL = Financial literacy
β5 TA = Technical aspect
β6 DEP = Dependency
β0:Intercept (constant term).
β1:(i = 1, 2, 3, 4, 5) Slope (coefficient of the independent variables).
ε:Error term (residuals).

According to the standard deviation-model summary estimation in Table 6, the first independent variable, awareness of online applications, is a significant predictor of users' adoption behaviour. The value of the users' adoption behaviour varies significantly. The regression model is statistically significant according to the F, p, and R^2^ values. The influence of awareness of online apps on users' adoption behaviour is substantial at the 5% limit for the p-value.

**Table 6:** Estimation of the standard deviation-Model Summary (Independent Variable-awareness of online applications), Theoretical Form of the Model, Users' adoption behaviour = a + b Awareness of Online Applications, Regression Model Summary^b^ 1.

Model	R	R Square	Adjusted R Square	Std. Error of the Estimate	F Change	Significance F
1	0.292	0.857	0.836	0.437	39.601	.000

a. Predictors: (Constant), Awareness of online applications.

b Dependent Variable: Users' adoption behaviour.

According to the standard deviation-model summary estimation in Table 7, the second independent variable, benefits & choice of investment, is a significant predictor of users' adoption behaviour. The value of the users' adoption behaviour varies significantly. The regression model is statistically significant according to the F, p, and R^2^ values. However, in the model, despite having a low R square and adjusted R square value, independent variables are statistically significant. This indicates the correlation between the independent variables and the dependent variable, but, they do not account for most of the variation in the dependent variable. The influence of benefits & choice of investment on user adoption behaviour is substantial at the 5% limit for the p-value.

**Table 7:** Estimation of the standard deviation- Model Summary (Independent Variable-benefits & choice of investment), Theoretical Form of the Model, Users' adoption behaviour = a + b Benefits & Choice of Investment Regression Model Summary^b^ 2.

Model	R	R Square	Adjusted R Square	Std. Error of the Estimate	F Change	Significance F
2	0.523	0.274	0.272	0.389	159.321	.000

a**.** Predictors: (Constant), Benefits & choice of investment.

b Dependent Variable: Users' adoption behaviour.

According to the standard deviation-model summary estimation in Table 8, the third independent variable, reliability, safety, and risk-related factors, is a significant predictor of users' adoption behaviour. The value of the users' adoption behaviour varies significantly. The regression model is statistically significant according to the F, p, and R^2^ values. However, in the model, despite having a low R square and adjusted R square value, independent variables are statistically significant. This indicates the correlation between the independent variables and the dependent variable, but, they do not account for most of the variation in the dependent variable. The influence of reliability, safety, and risk-related factors on user adoption behaviour is substantial at the 5% limit for the p-value.

**Table 8:** Estimation of the standard deviation- Model Summary (Independent Variable-reliability, safety, and risk-related factors) Theoretical Form of the Model, Users' adoption behaviour = a + b Reliability, safety, and risk-related factors, Regression Model Summary^b^ 3.

Model	R	R Square	Adjusted R Square	Std. Error of the Estimate	F Change	Significance F
3	0.587	0.345	0.343	0.370	222.366	.000

a**.** Predictors: (Constant), Reliability, safety, and risk-related factors.

b Dependent Variable: Users' adoption behaviour.

According to the standard deviation-model summary estimation in Table 9, the fourth independent variable, financial literacy, is a significant predictor of users' adoption behaviour. The value of the users' adoption behaviour varies significantly. The regression model is statistically significant according to the F, p, and R^2^ values. However, in the model, despite having a low R square and adjusted R square value, independent variables are statistically significant. This indicates the correlation between the independent variables and the dependent variable, but, they do not account for most of the variation in the dependent variable. The influence of financial literacy on users' adoption behaviour is substantial at the 5% limit for the p-value.

**Table 9:** Estimation of the standard deviation-Model Summary (Independent Variable-financial literacy) Theoretical Form of the Model, Users' adoption behaviour = a + b Financial literacy Regression Model Summary^b^ 4.

Model	R	R Square	Adjusted R Square	Std. Error of the Estimate	F Change	Significance F
4	0.149	0.022	0.020	0.452	9.700	.001

a**.** Predictors: (Constant), Financial literacy.

b Dependent Variable: Users' adoption behaviour.

According to the standard deviation-model summary estimation in Table 10, the fourth independent variable, the technical aspect, is a significant predictor of users' adoption behaviour. The value of the users' adoption behaviour varies significantly. The regression model is statistically significant according to the F, p, and R^2^ values. However, in the model, despite having a low R square and adjusted R square value, independent variables are statistically significant. This indicates the correlation between the independent variables and the dependent variable, but, they do not account for most of the variation in the dependent variable. The influence of technical aspects on user adoption behaviour is substantial at the 5% limit for the p-value.

**Table 10:** Estimation of the standard deviation- Model Summary (Independent Variable-technical aspect) Theoretical Form of the Model, Users' adoption behaviour = a + b Technical aspect, Regression Model Summary^b^ 5.

Model	R	R Square	Adjusted R Square	Std. Error of the Estimate	F Change	Significance F
5	0.284	0.080	0.078	0.438	37.049	.000

a. Predictors: (Constant), Technical aspect.

b Dependent Variable: Users' adoption behaviour.

According to the standard deviation-model summary estimation in Table 11, the sixth independent variable, dependency, is a significant predictor of users' adoption behaviour. The value of the users' adoption behaviour varies significantly. The regression model is statistically significant according to the F, p, and R2 values. The influence of dependency on the user's adoption behaviour is substantial at the 5% limit for the p-value.

**Table 11:** Estimation of the standard deviation- Model Summary (Independent Variable-dependency) Theoretical Form of the Model, Users' adoption behaviour = a + b Dependency, Regression Model Summary^b^ 6.

Model	R	R Square	Adjusted R Square	Std. Error of the Estimate	F Change	Significance F
6	0.801	0.642	0.641	0.273	758.831	.000

a**.** Predictors: (Constant), Dependency.

b. Dependent Variable: Users' adoption behaviour.

#### 5.2.5 Testing of hypotheses

According to the results of the regression, there were significant collective effects between the related factors, financial literacy, technical aspect, and dependency (R^2^ = 0.897, F (6, 359) = 287.099, p = .000). The relationship between the dependent and independent variables was determined by analyzing the predictors individually. The regression results indicated that “awareness of online applications” was a significant predictor in the model (R^2^ = 0.085, F (1, 423) = 39.601, p = .000); thus, H1 was accepted. In addition, “benefits & choice of investment” (R^2^ = 0.274, F(1, 424) = 159.321, p = .000) was found to be a significant predictor in the model; thus, H2 was accepted, “reliability, safety, and risk-related factor” (R^2^ = 0.345, F(1, 424) = 222.366, p = .000) was found to be a significant predictor in the model; thus, H3 was accepted, “financial literacy” (R^2^ = 0.022, F(1, 423) = 9.700, p = .001) was found to be a significant predictor in the model; thus, H4 was accepted, “technical aspect” (R^2^ = 0.080, F(1, 424) = 37.049, p = .000) was found to be a significant predictor in the model; thus, H5 was accepted, and “dependency” (R^2^ = 0.080, F(1, 423) = 37.049, p = .000) was found to be a significant predictor in the model; thus, H6 was accepted. An overview of the hypothesis testing is given in Table 12:

**Table 12:** Results of hypotheses testing.

Hypotheses	Regression Result	Result	Accept/Reject
H1: The adoption behaviour is influenced by users' awareness of such applications.	Significant	(0.000 < 0.05)	Accepted
H2: The users' adoption behaviour is influenced by the benefits and choice of investment of such applications.	Significant	(0.000 < 0.05)	Accepted
H3: Reliability, Safety, and risk-related factors significantly impact the users' adoption behaviour.	Significant	(0.000 < 0.05)	Accepted
H4: Financial literacy impacts the users' adoption behaviour.	Significant	(0.001 < 0.05)	Accepted
H5: The knowledge of technical aspects influences the users' adoption behaviour.	Significant	(0.000 < 0.05)	Accepted
H6: The users' adoption behaviour is influenced by the user’s investment dependency.	Significant	(0.000 < 0.05)	Accepted

Table 13 exhibits the ANOVA of the six regression predictor models, whereas Table 14 provides the coefficients of the regression models. The relationship between the models across all variables was evaluated using an ANOVA model. The ANOVA can assist in determining if the means of independent variables differ significantly. Once we realize that the means of each independent variable differ from one another, we may determine which independent variable is associated with our dependent variable.

**Table 13:** Variation analysis of the variables - ANOVA ANOVA.^a^

Model		Sum of Squares	Df	Mean Square	F	Sig.
1	Regression	7.576	1	7.576	39.601	.000^b^
	Residual	80.733	422	0.191		
	Total	88.309	423			
2	Regression	24.202	1	24.202	159.321	.000^c^
	Residual	64.106	422	0.151		
	Total	88.309	423			
3	Regression	30.475	1	30.475	222.366	.000^d^
	Residual	57.834	422	0.137		
	Total	88.309	423			
4	Regression	1.984	1	1.984	9.700	.001^e^
	Residual	86.325	422	0.204		
	Total	88.309	423			
5	Regression	7.127	1	7.127	37.049	.000^f^
	Residual	81.182	422	0.192		
	Total	88.309	423			
6	Regression	56.749	1	56.749	758.831	.000^g^
	Residual	31.559	422	0.074		
	Total	88.309	423			

a Dependent Variable: Users' adoption behaviour.

b Predictors: (Constant), Awareness of online applications.

c Predictors: (Constant), Benefits & choice of investment.

d Predictors: (Constant), Reliability, Safety, and risk-related factors.

e Predictors: (Constant), Financial literacy factors.

f Predictors: (Constant), Technical aspect factors.

g Predictors: (Constant), Dependency factors.

**Table 14:** Coefficients regression models 1, 2, 3, 4, 5, and 6.

Coefficients^a^					
Model	Unstandardized Coefficients		Standardized Coefficients	t	Sig.
	B	Std. Error	Beta		
(Constant)	0.668	0.089		7.513	.000
Awareness of online applications	−0.111	0.028	0.215	6.292	.000
Benefits & choice of investment	0.215	0.020	0.379	12.622	.000
Reliability, Safety, and risk-related factors	0.159	0.025	0.506	14.911	.001
Financial literacy	−0.186	0.023	0.090	3.114	.000
Technical aspect	0.105	0.021	0.162	6.086	.000
Dependency	0.668	0.023	0.742	27.546	.000

a Users' adoption behaviour.

The values of coefficients regression of all six variables are given in Table 14. According to the values, the sixth predictor has the highest impact on the user’s adoption behaviour (β = .742, t = 27.546, p < .05), followed by the impact of the third predictor (β = 0.506, t = 14.911, p < .05), second predictor (β = 0.379, t = 12.622, p < .05), first predictor (β = 0.215, t = 6.292, p < .05), fifth predictor (β = 0.162, t = 6.086, p < .05), and fourth predictor (β = 0.090, t = 3.114, p < .05) respectively.

## 6 Results and discussions

This study investigates the influence of awareness, benefits & choice of investment, reliability, safety, risk-related factors, financial literacy, technical aspect, dependency, and adoption behaviour on online stock trading applications. Based on the study's findings, it has been determined that these variables significantly impact the use of online stock trading applications. According to descriptive statistics analysis, the use of such applications is significantly affected by all six independent factors.

The mean value of each variable is close to the fourth Likert point scale. As a result, it suggests that all variables are at “agree” levels. The most significant standard deviation, which reflects the use of online stock trading applications, is 0.799. Conversely, dependency has the lowest standard deviation at 0.493%. Cronbach's alpha analysis revealed that the internal consistency and dependability of the data were extremely high. According to the results of the correlation research, there was a strong and positive link between the variables. According to regression results, all six variables considerably impact the use of online stock trading applications.

People are aware of the many online trading applications and software programs accessible for stock trading, according to the findings of the survey. They are familiar with the method for opening online trading apps for stock trading and are knowledgeable about the proper usage of such an application in the market. Authors revealed that investors collect stock information from numerous sources such as journals, financial markets, newspapers, social media, the Internet and broking firms' websites and current update published by National Stock Exchange and Bombay Stock Exchange from time to time [51]. People can have updated information about stock market awareness and investment [7].

Their stock market investment preferences influence individuals' awareness of Internet trading programs. People's awareness of Internet trading applications is influenced by their positive or negative views about stock market investing. The Internet has given a new edge to businesses to transform from traditional to digital [52], and online mobile trading and investment applications have changed the face of the Indian stock market. Online trading programs also facilitate instantaneous transactions.

Users believe that trading through available online trading applications for stock trading is safe and more reliable. There is no danger involved in trading stocks using online trading apps. People also believe that online trading applications always provide a cyber-security risk that might result in phishing, hacking, and cyber-attacks [32,34,38]. The authors identified that people use the licensed firewall security app for online payment [53]. Stock trading via Internet trading programs encourages green investing and adds to sustainable financing [54].

The study highlighted the importance of financial literacy when utilizing such tools. People believe financial literacy influences their decisions to select a productive portfolio through online trading tools. In addition, they affirm that they need to acquire more expertise before being permitted to choose the best stocks for trading using an online trading application. Knowledge of buying and selling shares through online trading applications and the technical and financial aspects of stock trading when utilizing such online trading applications influence the use of such programs. Authors claimed that knowledge and analyzing skills of financial products and services are required to understand the investors to minimize the risk of financial services and products [18].

Awareness of the technical component was also identified as a significant factor influencing the utilization of online trading applications. Knowledge of the algorithm used for stock trading and familiarity with all the technical aspects of online stock trading programs are critical obstacles to using these applications.

The study's findings revealed that individuals think using an online trading application is a practical approach to trading stocks. It contributes to raising interest in investing in the stock market. Using an online trading application can save time. Adopting an Internet trading program reduces reliance on a stockbroker or financial advisor. Prospective investors gain trading independence by utilizing an Internet trading platform. Using an Internet trading program is more affordable and cost-effective. Adopting and using online trading software boosts one's financial and technical understanding of the stock market.

## 7 Conclusion, limitations, and future scope

With the help of stock market applications, everyone may trade and invest. These intricate processes are automated, traders are given useful financial tools, and participation risks are reduced. The study aims to analyze the factors associated with using such applications by users. Some important implications may be drawn from the study's findings and analysis. Based on the descriptive analysis, it is evident that all factors significantly impact the user's adoption behaviours. According to the results of the correlation analysis, a significant association was found between the variables. The results of regression models concluded that all six variables significantly influence the users' adoption behaviour. This signifies the significant impact of users' awareness of online applications, benefits & choice of investment, reliability, safety, risk-related factors, financial literacy, technical aspect, and dependency on adopting these online share trading applications.

The study's main limitation is that it primarily focuses on the user's adoption behaviour on online stock applications, whereas the other modes of investment have not been considered. Future research should consider the impact of these variables in all modes of stock market investments.

The stock market alleviates cash constraints, fostering economic expansion. The stock market's liquidity decreases investment risks since investors can alter their portfolios to avoid potential losses. This makes investment more enticing since individuals may now exercise control. This study will contribute significantly to understanding the behavioral aspects of the stock market application and its uses. The findings will be helpful to the prospective investor in understanding the factor affecting the adoption of such applications and the companies involved in running or making such applications to understand users' behaviour.

## Author contribution statement

Amar Johri: Mohamma Wasiq: Harpreet Kaur: Mohammad Asif: Conceived and designed the experiments; Performed the experiments; Analyzed and interpreted the data; Contributed reagents, materials, analysis tools or data; Wrote the paper.

## Data availability statement

Data will be made available on request.

## Declaration of competing interest

The authors declare that they have no known competing financial interests or personal relationships that could have appeared to influence the work reported in this paper
