# Supplementary material for: Assessment of users' adoption behaviour for stock market investment through online applications
Source: Heliyon. 2023 Aug 29;9(9):e19524. doi: 10.1016/j.heliyon.2023.e19524 (PMC10558715; doi:10.1016/j.heliyon.2023.e19524)
Supplement: Multimedia component 1 [file mmc1.docx]

**Questionnaire**

**Stock Market Investment through Online Applications**

Investing in stocks has become increasingly accessible, with beginners able to start an account via a brokerage's website or mobile app with very little money. The present study is aimed at examining the investor’s perception and adoption of online applications for stock market investment.

**Demographic Profile of the Respondents**

**Age**

- Below 20
- 21-30
- 33-40
- 41-50
- 51-60
- Above 60

**Gender**

- Male
- Female

**Profession**

- Student
- Private Employee
- Government Employee
- Business

**Monthly Income Level**

- 20,000-40,000
- 40,000-60,000
- 60,000-80,000
- 80,000-100,000
- Above 100,000

**Do you invest in the stock market?**

- Yes
- No

**If yes then, which one of the following modes do you prefer to invest in the stock market?**

- Financial Advisor
- Online brokerage account/ Online Apps (Invest on your own)

**If no then, which one of the following modes will you prefer to invest in the stock market?**

- Financial Advisor
- Online brokerage account/ Online Apps (Invest on your own)

**Statement Based Questions based on a five-point Likert scale**

Strongly Agree = 5

Agree = 4

Neutral = 3

Agree = 2

Disagree = 1

1. You are aware of the various online trading applications available for stock trading.
2. You are aware of the different options of the software application available for stock trading.
3. You are aware of the procedure for opening online trading applications available for stock trading.
4. You have all the information about the proper use of such an application for stock trading in the market.
5. Your awareness of online trading applications is affected by your choice of investment toward investing in the stock market.
6. Your awareness of online trading applications is affected by your positive or negative perceptions of investing in the stock market.
7. Online trading applications provide the benefits of immediate transactions.
8. It is safe to do trading through online trading applications available for stock trading.
9. It is more reliable to trade through online trading applications available for stock trading.
10. There is no risk of trading in the stock market through online trading applications.
11. Online trading applications always carry a risk of cyber security that leads to phishing, hacking, and cyber-attacks.
12. Stock trading with the help of online trading applications contributes to sustainable financing and promotes green investment.
13. You are financially literate and confident enough to choose a portfolio that will be profitable on your own through online trading applications.
14. You have enough knowledge to choose the right stocks for trading with the help of an online trading application.
15. You are aware of all technical and financial aspects of stock trading while using such online trading applications.
16. You have all the knowledge about the process of buying and selling shares through online trading applications.
17. You are aware of the algorithm used for stock trading.
18. You are aware of all the features of your online stock trading application.
19. You have better control of stock trading through these online trading applications.
20. Stock trading through online trading applications of your own decreases your dependency on trading through middlemen.
21. You feel comfortable accessing these software applications for stock trading.
22. Stock trading through an online trading application provides monitoring of investment anytime.
23. Adopting an online trading application is a convenient way to do stock trading.
24. Adopting an online trading application helps in increasing the interest in stock market trading.
25. Adopting an online trading application is time-saving.
26. Adopting an online trading application decreases the dependency on a financial advisor/stockbroker.
27. Adopting an online trading application gives you trading freedom.
28. Adopting an online trading application is cost-effective and cheaper.
29. Adopting an online trading application and the use of such applications increases financial and technical awareness of the stock market.
